# What to do about hepatocellular carcinoma: Recommendations for health authorities from the International Liver Cancer Association

**DOI:** 10.1016/j.jhepr.2022.100578

**Published:** 2022-09-08

**Authors:** Manon Allaire, Jordi Bruix, Marko Korenjak, Sarah Manes, Zorana Maravic, Helen Reeves, Riad Salem, Bruno Sangro, Morris Sherman

**Affiliations:** 1 AP-HP Sorbonne Université, Hôpital Universitaire Pitié-Salpêtrière, Service d’Hépato-gastroentérologie, Paris, France; 2 University Hospital Clinic IDIBAPS, Barcelona, Spain; 3 European Liver Patients' Association (ELPA), Brussels, Belgium; 4 Global Liver Institute Washington District of Columbia, USA; 5 Digestive Cancers Europe, Brussels, Belgium; 6 The Newcastle University Centre for Cancer, Newcastle University, Newcastle upon Tyne, UK; 7 Department of Radiology, Section of Interventional Radiology, Department of Radiology, Northwestern Memorial Hospital, Chicago, IL 60611, USA; 8 Liver Unit and HPB Oncology Area, Clinica Universidad de Navarra and CIBEREHD, Pamplona, Spain; 9 University Health Network, Toronto, Ontario, Canada

**Keywords:** Hepatocellular carcinoma, Viral hepatitis, Alcohol consumption, Obesity, Hepatocellular carcinoma surveillance, Hepatocellular carcinoma treatment, AASLD, American Association for the Study of Liver Disease, AFP, alpha-fetoprotein, ALT, alanine aminotransferase, APRI, aspartate aminotransferase-to-platelet ratio index, BCLC, Barcelona clinic liver cancer, cTACE, conventional TACE, DCP, des-gammacarboxy prothrombin, DEB-TACE, TACE with drug-eluting beads, EASL, European Association for the study of the Liver, EBRT, external beam radiation therapy, ELF, enhanced liver fibrosis, GGT, gamma-glutamyltransferase, HCC, hepatocellular carcinoma, Li-RADS, Liver Imaging Reporting and Data System, NAFLD, non-alcoholic fatty liver disease, RFA, radiofrequency ablation, TACE, transarterial chemoembolisation, TARE, transarterial radioembolisation, TKI, tyrosine kinase inhibitor

## Abstract

Hepatocellular carcinoma (HCC) is a major public health problem worldwide for which the incidence and mortality are similar, pointing to the lack of effective treatment options. Knowing the different issues involved in the management of HCC, from risk factors to screening and management, is essential to improve the prognosis and quality of life of affected individuals. This document summarises the current state of knowledge and the unmet needs for all the different stakeholders in the care of liver cancer, meaning patients, relatives, physicians, regulatory agencies and health authorities so that optimal care can be delivered to patients. The document was commissioned by the International Liver Cancer Association and was reviewed by senior members, including two ex-presidents of the Association. This document lays out the recommended approaches to the societal management of HCC based on the economic status of a given region.

**Key points**

• Hepatocellular carcinoma (HCC) is an under-recognised health threat.
• The underlying liver diseases that predispose to HCC are frequently not diagnosed.
• Among those whose liver disease has been diagnosed, there is insufficient recognition of who is at risk of developing HCC.
• There are many HCC risk scores that could help to identify who is at risk of HCC.
• Even when risk is recognised, early detection programmes are seldom instituted and, when instituted, are poorly adhered to.
• The availability of appropriate and effective treatment is highly variable in high-resource regions and frequently not available at all in low-resource regions.
• The societal management of HCC requires health authorities to institute appropriate programmes.
• HCC is best managed in expert centres.

## Introduction

It is important that the different stakeholders in the care of liver cancer have knowledge of the disease, including its prognosis and management. This document summarises the current state of knowledge and unmet needs and is expected to generate awareness for patients, relatives, physicians, regulatory agencies and health authorities to ensure optimal care, shared decision-making (between patients and care providers), and enhanced compliance and quality of life. This document first describes the epidemiology of the underlying liver diseases that lead to HCC. This is followed by a description of the different stages of disease management, including primary prevention of liver disease, diagnosis of liver disease and its severity, surveillance for HCC, investigation of suspected HCC and management possibilities. Then follows a discussion of potential approaches in regions of the world with different healthcare resources, and finally some thoughts about future research.

This document should be read as a companion piece to the European Association for the study of the Liver (EASL)’s demands of European health authorities regarding HCC care and as a counterpoint to the document on cancer screening produced by the European Union, which did not mention HCC at all.

The reasons why HCC warrants special attention from the public and from medical authorities are listed in Box 1.

Box 1 **Why HCC should warrant special attention from the public and from medical authorities.**

**Figure:**
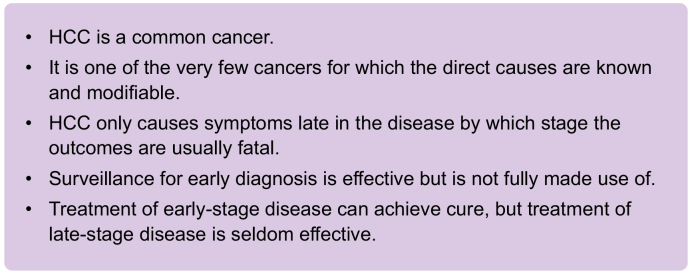

HCC, hepatocellular carcinoma.

For the majority of cases of HCC, the risk factors that precede the development of the cancer and the causes of these risks are well known.^1^^,^^2^ These include chronic viral hepatitis, moderate to heavy chronic alcohol use, diabetes and obesity, and smoking. Smoking is not a direct cause but contributes to risk when other risk factors are present. Other less common causes of HCC, such as genetic haemochromatosis or alpha 1-antitrypsin deficiency are not discussed in this document but many of the recommendations pertinent to other causes of HCC will apply to these conditions as well. Treatment of the causes of the underlying liver disease as well as lifestyle adaptations may reduce the occurrence of liver cancer. Even if the role of diet is not fully established, coffee may have a preventive effect.^3^ Many herbal medicines, such as aristocholic acid, that are available, are harmful to the liver and should not be used.^4^ These findings suggest that primary prevention of HCC is feasible. Since treating established HCC is expensive, prevention is likely to provide the greatest benefit at the lowest cost, which will be particularly relevant for countries with more limited healthcare resources (see Box 2).

Box 2 **Initiatives for government entities to reduce obesity in childhood.11**

**Figure:**
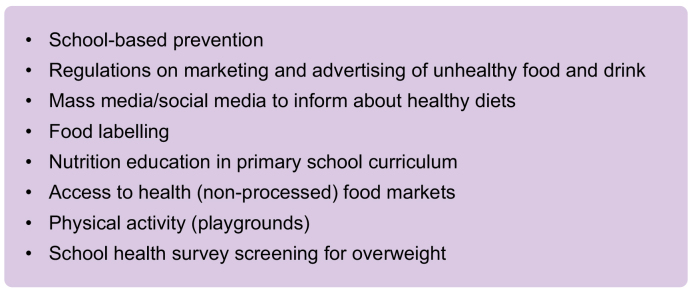

All the causes of HCC listed above induce chronic inflammation in the liver. Over time inflammation causes necrosis (death of cells). This heals by laying down scar tissue, known as fibrosis. Over time fibrosis accumulates in specific patterns to form nodules of liver tissue surrounded by fibrosis. This is cirrhosis. The many rounds of necrosis and regeneration induced by inflammation also predisposes to mutations in the genome of liver cells, and over time sufficient mutations accumulate to cause the cells to become malignant. Thus, HCC arises as a consequence of the chronic inflammation, but the marker of this process is fibrosis. At-risk individuals can be identified by determining the extent of fibrosis, or using several risk scores which are based on age, sex, predisposing cause and severity of liver fibrosis.

Currently, most HCCs even in high-resource regions present as late-stage disease that is frequently not curable, and often not even suitable for palliative systemic therapy. Yet there is potential to change this, even in low-resource regions, by introducing early detection programmes based on readily available tools. HCC surveillance has the potential to uncover lesions that are amenable to cure. The methods available to effect cure range from highly sophisticated to fairly low tech; results vary accordingly, but even low-tech methods have the potential to reduce the death rate.

HCC is one of the few cancers for which the incidence and mortality are similar. Systemic agents currently in use are not curative but can prolong life. Even when response to treatment is achieved, long-term survival is limited by the impairment of underlying liver function. Indeed, an additional level of complexity when treating liver cancer, as opposed to virtually any other cancer, is that inherent liver dysfunction limits the treatments that can be offered. As cirrhosis progresses it replaces functioning liver tissue with scar tissue. Initially liver function is preserved, but over time liver function deteriorates as more and more functioning tissue is replaced. Decreased liver function may preclude any form of therapy as poor liver function confers a major risk of developing irreversible liver failure due to the destruction of remaining functioning liver tissue that accompanies attempts at cure.

## Epidemiology of HCC

Globally, HCC is the 6^th^ most common cancer in incidence and ranks 3^rd^ in mortality rate.^5^ There are significant regional differences with the highest incidence being in Sub-Saharan Africa, China and neighbouring countries in South East Asia (Table S1).^6^ However, the epidemiology is changing, and those regions where HCC has the highest incidence are recording falling incidence rates. In the United States, the incidence has been decreasing since 2011, probably related to control of viral hepatitis. Those regions that are considered to be low incidence regions are documenting increases in incidence, in some cases of more than 20% over the period of 1990–2015.^7^^,^^8^ Furthermore, the prevalence of the causes of the underlying liver disease varies by region, with hepatitis B being the most common cause in China, South East Asia and sub-Saharan Africa, while alcohol is the most common cause in Europe.^6^ In the high-income Pacific region (Japan, Australia and New Zealand), hepatitis C is the most common underlying cause, as is the case in North Africa and the Middle East.^6^ Non-alcoholic fatty liver disease (NAFLD) is not the largest contributor to HCC anywhere, but in North America and in Oceania more than 20% of cases are related to NALFD.^6^ Overall about 33% of cases are due to hepatitis B, 21% to hepatitis C, 30% to alcohol-related liver disease and about 16% to other causes, of which NAFLD is the most common.^6^ About 72.5% of cases occur in Asia, 9.7% in Europe, 7.8% in Africa, 5.1% in North America and 4.4% in Latin America and the Caribbean.

### Hepatitis B

There are an estimated 257 million people with chronic hepatitis B of whom it is estimated that no more than 11% have been diagnosed and no more than 17% of those (about 5 million) have been treated.^9^ Hepatitis B is a blood borne disease, that is also transmitted sexually. The major route of transmission prior to the introduction of neonatal vaccination was to infants from other infected family members, commonly the mother, but other family members may also transmit the virus. Infection with hepatitis B in early childhood tends to lead to chronic life-long infection, whereas infection in adolescence or adulthood tends to cause an acute usually self-limited infection. Transmission in infancy and early childhood therefore accounts for most cases of chronic hepatitis B. Sexual transmission among adolescents and adults is largely between a chronic carrier and an uninfected individual. Newly infected adults with acute hepatitis B may be ill and thus unlikely to engage in sexual activity, and in addition, the infectious period is brief in individuals with acute self-limited infection, making spread much less likely than from a chronic carrier. Spread by transfusion or contaminated medical equipment is an uncommon to rare method of transmission today. Regions of high hepatitis B prevalence are shown in Table S2.^9^

### Hepatitis C

Hepatitis C is parenterally transmitted. Sexual transmission is uncommon. Although initially discovered in transfusion recipients and subsequently in injection drug users, globally the most common route of transmission was, and is, from improperly sterilised re-usable needles and syringes.^9^ Treatment for schistosomiasis by injection was responsible for the very high prevalence of chronic hepatitis C in Egypt, while other similar campaigns, such as treatment programmes against malaria in Cameroon and Gabon,^10^ have also led to the rapid spread of hepatitis C. Today, many new infections with hepatitis C are related to injection drug use or tattooing procedures (*e.g.*, in prisons), but in some countries non-disposable needles and syringes are still used for medical procedures. It is estimated that globally about 5% of injections are still unsafe.^9^ Overall, in 2015 there were about 1.75 million new hepatitis C infections. While in 2015, it was estimated that only about 20% of hepatitis C-infected individuals had been diagnosed.^9^ More recent data is not available. Regions of high hepatitis C prevalence are shown in Table S3.^9^

### Non-alcoholic fatty liver disease

NAFLD is largely a consequence of modern Western diets that contain excess sugars and fats. Thus, NAFLD is most frequently seen in overweight individuals. Type 2 diabetes mellitus is also associated with NAFLD, and it is probable that diabetes *per se* is also a cause of NAFLD independent of the effects of obesity.

**Fig. 1:**
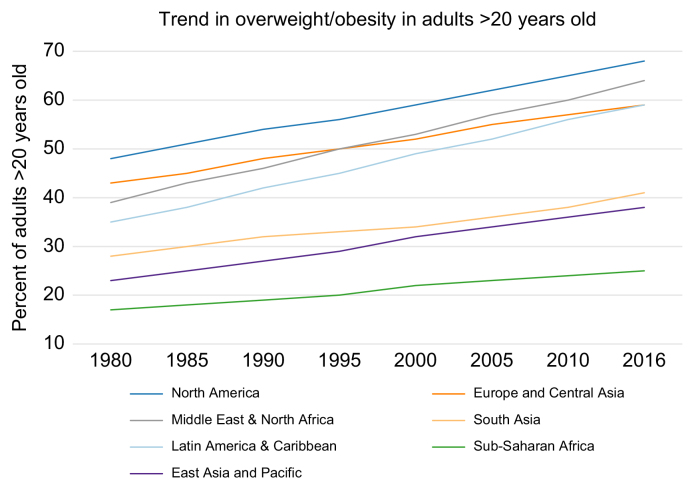
Trends in overweight/obesity in adults >20 years old by geographical region. Adapted from.^11^

Obesity is found in all societies, but with increasing adoption of a Western diet and a sedentary lifestyle, data shows that obesity rates are rising in virtually every country in the world.^5^ Trends in obesity over time are shown in Fig. 1.^11^ Table 1 lists some of the regions with the highest obesity rates.^11^ In addition, sedentary lifestyle, obesity, diabetes and inadequate diet often overlap with alcohol consumption, which further increases the risk of liver disease and HCC.

**Table 1:** **Percent of population that is obese in selected countries**.^11^

Region/country	Obese (%)	World rank
Pacific Islands	46-61	1-10
USA	36.2	12
Canada	29.4	26
Hungary	26.4	42
Spain	23.8	62
Russia	23.1	70
Germany	22.3	79
France	21.6	87
Italy	19.9	107

### Alcohol-related liver disease

**Fig. 2:**
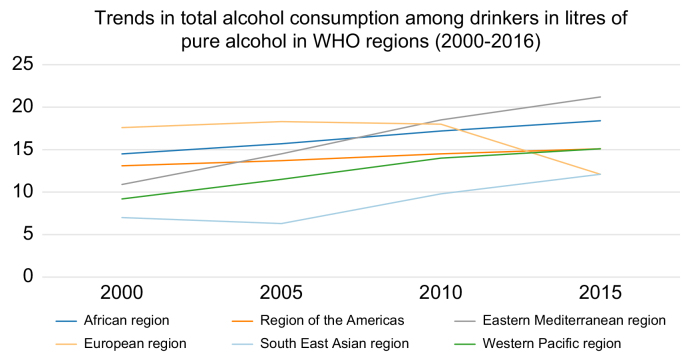
Trends in total alcohol consumption between 2000–2016 by WHO region. Adapted from.^12^

The link between excess alcohol consumption and cirrhosis is well known. Alcohol-related cirrhosis is common and since HCC is associated with cirrhosis, excess alcohol consumption is a direct cause of HCC, although globally less common than viral hepatitis. Nonetheless, in some parts of the world, where alcohol consumption is very high (*e.g*., Hungary, France, Russia) alcohol-related liver disease is one of the most common causes of HCC. In the WHO regions of Africa, the Americas, the Eastern Mediterranean and European regions, the percentage of people who use alcohol has declined since 2000. However, it has increased in the Western Pacific Region and has remained stable in the South East Asia Region.^12^ Fig. 2 shows changes in alcohol consumption over time in different World Health Regions.^12^ Overall, the attributable fraction is about 26%, *i.e.* in the absence of alcohol consumption there would be 26% fewer HCCs.^13^

All this data indicates that risk factors for HCC are widely present and persistent, and as such are targets for intervention.

## Reducing HCC-related mortality

HCC-related mortality can be reduced substantially. The approach has five main pillars:

• Prevention of liver disease.
• Recognising liver disease in individual patients.
• Recognising who among those with liver disease are at risk of developing HCC.
• Providing surveillance to those who are at significant risk.
• Providing treatment at a stage when cure is still possible. (Providing treatment for those with incurable disease is necessary but will not decrease mortality).

### Prevention of liver disease

#### Prevention of liver disease by control of hepatitis B infection

Worldwide, hepatitis B remains the most common cause of HCC. Vaccination against hepatitis B will protect upcoming generations. There is incontrovertible evidence from the universal neonatal vaccination programme in Taiwan and elsewhere that hepatitis B vaccination results in a reduction in HCC incidence.^14^^,^^15^ Many prevalence surveys on children born before and after the Taiwanese national hepatitis B vaccination programme began showed a 75% decrease in the incidence of HCC in children 6-9 years of age. This benefit was sustained for at least 20 years. Many economic studies have shown that under most assumptions neonatal vaccination for hepatitis B is actually cost saving.^16^^,^^17^

Universal adoption of hepatitis B vaccination has the potential to cause the greatest reduction in global HCC incidence and therefore mortality. Protection from hepatitis B by vaccination will also protect against hepatitis D.

Clinical guidelines also indicate that family members and close contacts of a hepatitis B-infected individual should be screened for the disease. Furthermore, where hepatitis D is prevalent those infected with hepatitis B should also be tested for hepatitis D.

Most countries have instituted universal neonatal vaccination, but in some Western countries universal adolescent vaccination has been introduced with maternal hepatitis B screening and vaccination of their newborns. The rationale for adolescent vaccination is not clear. First, this strategy ignores the role of other family members besides the mother as sources of infection, and second, the source of most transmission of hepatitis B is from chronic hepatitis B carriers, and most chronic carriers acquire their disease in infancy or childhood. Since immigration from regions of high HBV endemicity to regions of low endemicity is frequent (*e.g*., from China to North America) even jurisdictions with nominally low hepatitis B endemicity should institute universal infant vaccination in order to decrease the risk of those individuals infected in their native countries from spreading the disease to their offspring.

Effective hepatitis B treatment is available. Furthermore, testing for hepatitis B costs about $0.50/test, making it economical to test broadly.^9^ Treatment with tenofovir derivatives or entecavir will suppress viral replication although they will not eradicate the virus. Nonetheless, long-term treatment with these agents will result in sustained viral suppression and resolution of hepatic inflammation. Fibrosis may also resolve but, if cirrhosis was present prior to the institution of antiviral therapy, the risk of cancer remains, although it decreases with time. For example, one study showed a reduction in HCC incidence from 20% to 5% after 8 years of viral suppression.^18^ Tenofovir disoproxil fumarate (but not tenofovir alafenamide) and entecavir are no longer patent protected and inexpensive generic versions are available.

Treatment of active hepatitis B that is initiated before the onset of cirrhosis virtually guarantees that HCC will not develop. Suppression of viral replication causes a reduction or even elimination of inflammation in the liver, thereby breaking the chain of events that lead to HCC. Resistance to these agents is uncommon to rare.

Thus, the means exist to prevent and treat hepatitis B and thereby reduce the most common cause of HCC.

#### Prevention of liver disease by control of hepatitis C infection

The route of spread of hepatitis C is well known and easily prevented by avoiding re-use of injection needles and syringes. In the healthcare setting this is easily managed if resources are available, but in other scenarios (*e.g.*, injection drug use, tattoos in prisons) this is less easily accomplished. In some parts of the world tribal rituals involve scarification or other procedures that break the skin (*e.g*., tattooing among the Māori or puberty rites in some African tribes). This is another route of infection of both hepatitis B and C.

For those already infected, very effective hepatitis C treatments are available that virtually guarantee that HCC will not develop if given early enough. Several combinations of nucleoside analogues, *e.g*. sofosbuvir/ledipasvir or glecaprevir/pibrentasvir can be used. These combinations are effective against all genotypes of hepatitis C and the development of resistance to the combinations is rare.^9^ Even once cirrhosis has developed, treatment with these regimens decreases the HCC risk,^19^^,^^20^ although a reduction in incidence in individuals with HCV may not be apparent in the first 5 years of follow-up.

Because hepatitis C infection itself does not cause symptoms, the disease frequently only manifests with the complications of cirrhosis or with HCC. Therefore, in order to reduce hepatitis C-related HCC, infected individuals need to be identified and referred for treatment. Since treatment of hepatitis C infection results in cure, *i.e.* elimination of the virus, hepatitis C as a disease can theoretically be eradicated, but to do so will require investment in programmes such as those instituted in Egypt where hundreds of thousands of people have been identified and treated in a relatively short time. Apart from Egypt, few countries have instituted screening programmes to detect infected individuals. In Western countries, the highest prevalence of hepatitis C is in the so-called baby boomer generation. Cost-efficacy analyses show that one-time screening of baby boomers (born between 1945 and 1975 or 1985 would be cost effective and would identify about 70-80% of those infected with hepatitis C^21^). Testing for elevated alanine aminotransferase (ALT) and follow-up as part of routine medical practice will identify additional cases. Earlier identification and treatment of individuals with hepatitis C will reduce HCC incidence. Outside Western countries, different population screening targets may be more appropriate.

Therefore, as with hepatitis B, the means exist to interrupt hepatitis C transmission and cure those currently infected, thereby eliminating the second most common cause of HCC.

#### Prevention of liver disease by control of NAFLD

Most factors resulting in obesity and NAFLD are cultural, *e.g*. Western diets. This makes reducing the incidence of NAFLD-related HCC much more difficult than for viral hepatitis. NAFLD is, however, reversible with lifestyle-induced weight loss.^22^ The difficulty is in getting patients to adhere to diet and exercise requirements. More than 75% of overweight and obese individuals live in middle-income regions.^11^ Over time the burden of obesity has increasingly fallen on the poor, including in wealthy countries.^11^

In the short term, a reduction in NAFLD-related HCC is probably not a realistic goal. In the longer-term such a reduction will require measures to tackle obesity (see later).

#### Prevention of liver disease by control of alcohol-related liver disease

Attempting to reduce the incidence of HCC by reducing the prevalence of alcohol-related liver disease will require a whole-of-society approach. Although alcohol consumption is rising globally, Fig. 2 shows that in some regions consumption is actually declining slightly or remaining stable.^12^ There are some strategies that have been associated with a reduction in alcohol consumption, such as taxing alcohol heavily, but in addition, major cultural shifts are necessary to reduce the glamorisation of excessive drinking. Just as the depiction of smoking in film and TV has become noticeable by its absence, the depiction of drinking, either social or excessive, should be limited. There are methods to reduce alcohol consumption that have been implemented in various jurisdictions, with some success. However, these methods require government action as well as involvement of various levels of society (see later).

### Prevention of death from HCC by identifying existing liver disease

Identifying liver disease early provides an opportunity to treat the liver disease, if that is possible, and to provide surveillance for HCC to detect early-stage curable disease.

Chronic liver inflammation is asymptomatic. Symptoms only develop once liver function has deteriorated. However, because the liver has a large reserve capacity, symptoms due to decreased liver function only occur late in the course of all chronic liver diseases, once the liver reserve capacity has been exhausted. Thus, with all chronic liver diseases there is a long sub-clinical phase when intervention has the best chance of preventing liver failure and HCC.

Despite not causing symptoms, liver disease can be diagnosed early, well before the risk of complications starts to rise. Hepatic inflammation causes blood test abnormalities, mainly an elevation of ALT and gamma-glutamyltransferase (GGT), which are made in the liver and released into the blood stream by injured or dying liver cells. These tests are commonly measured in the blood and are part of the panel of tests that are routinely used by family practitioners at the annual or bi-annual check-up. Although the tests are performed frequently, minimal elevations of ALT and GGT are often ignored, because there may be no other evidence of liver disease. This constitutes a missed opportunity because any level of this test that is above the normal range is specific for liver disease. However, it should be noted that the commonly used upper limit of the normal range quoted by laboratories is too high^23^^,^^24^ and liver disease can be present even with apparently normal transaminase levels. Of course, if liver disease is diagnosed, additional testing is required to identify the cause, but this discussion is beyond the scope of this document.

Assuming that liver disease is recognised as being present, the next step is to determine the severity of disease, in particular, how much fibrosis is present in the liver. The easiest way of doing this is with either a panel of blood tests such as Fibrotest, the enhanced liver fibrosis (ELF) score, the aspartate aminotransferase-to-platelet ratio index (APRI) panel or similar tests (Table S4),[25], [26], [27], [28], [29], [30], [31] or by measurement of liver stiffness by transient or shear-wave elastography,^32^ or less conveniently, a biopsy of the liver. Standard liver blood tests such as albumin, bilirubin and international normalised ratio do not assess the degree of fibrosis. Abnormalities of these tests indicate liver dysfunction. However, the objective is to diagnose liver disease before these tests become abnormal. Another simple method of assessing whether cirrhosis is present is to measure the platelet count. A decreased platelet count in the presence of known liver disease, and assuming no haematological disorder, is indicative of at least moderately advanced cirrhosis. The panels of blood tests for fibrosis were developed in comparison to liver biopsy, which is an imperfect “gold standard”. Since the various blood test panels do not have a one-to-one relationship with liver biopsy, they may have a significant error rate. Thus, at best, the fibrosis blood test panels give a rough idea of the amount of fibrosis present.

Shear-wave elastography is a form of ultrasound that bounces ultrasonic waves off the liver and measures how “wobbly” the liver is. The more fibrosis, the less “wobbly”. Thus, for most cases it is possible to get an assessment of the severity of liver disease without resorting to biopsy. In the absence of significant hepatic inflammation a liver stiffness value <10 kPa precludes advanced chronic liver disease whereas a value ≥25 kPa suggests advanced chronic liver disease with clinically significant portal hypertension and is thus associated with a higher risk of liver decompensation.^33^ Severity of fibrosis is graded from stage 0 (no fibrosis) to 4 (cirrhosis). Stages 3 and 4 are considered to represent advanced fibrosis.

Thus, the possibility exists, using routine, widely available tests, to diagnose liver disease and assess liver disease severity well before the onset of symptoms, at a time when the risk of developing HCC is low to non-existent.

### Assessing HCC risk

Not all individuals with liver disease, or even cirrhosis, will develop HCC. HCC can develop on a non-cirrhotic liver, although this is much less common and affected individuals would usually present with at least stage 3 fibrosis (major fibrosis without complete cirrhosis) or resolved cirrhosis. The exception to this is the fibrolamellar variant of HCC, which develops on the background of a histologically normal liver. This entity is uncommon to rare, related to a specific genomic abnormality and is not discussed here. Resolved cirrhosis is seen mainly in those who have been successfully treated for hepatitis B and hepatitis C. In the absence of continued inflammation much of the fibrosis is resorbed. There are histological markers of this state, but the findings are subtle. In these patients the risk for HCC declines with time. However, it is currently unclear whether the risk ever approaches the risk in a non-cirrhotic population, nor is it clear at what point surveillance would no longer be useful.

Risk scores have been developed that help to identify those who are at a significant risk of developing HCC. Most use readily available test results and demographic details to assess risks. None of these tests have very high accuracy, but they are perhaps more useful for determining those who are not at risk and therefore do not need surveillance. Some of these scores and the populations in which they were developed are described in Table S5.[34], [35], [36], [37], [38], [39], [40], [41], [42], [43], [44], [45] If used, the scores should be restricted to populations similar to those in which the scores were initially developed or validated. In regions where testing for fibrosis stage is available, risk scores are probably of little benefit for early-stage fibrosis. However, where testing for fibrosis stage is not available, it may be that all patients known to have liver disease regardless of clinical evidence of cirrhosis may benefit from a risk score to determine who should and should not undergo surveillance.

Thus, HCC risk can be easily determined. This should be routine in all individuals with liver disease.

### Surveillance for HCC

HCC surveillance results in the detection of small lesions that are amenable to potentially curative therapy more often than in the absence of surveillance. However, surveillance for HCC has not been subject to rigorous randomised testing to determine whether it results in fewer deaths. Nonetheless, there are multiple lines of evidence that this is indeed the case. The simplest evidence is that presentation of HCC with symptoms is seldom curable and seldom even treatable, whereas early-stage disease is frequently curable. Multiple studies have suggested that surveillance does improve survival. What is at issue in these studies is whether the lead time bias, length bias and overdiagnosis bias inherent in these studies is sufficient to negate the apparent improvement in survival. Studies that take lead time into account suggest that survival is prolonged although mainly with more rapidly growing tumours.^46^^,^^47^

This represents an opportunity for public health officials – even though surveillance is effective in finding small curable HCCs, it is neither widely practiced by healthcare professionals, nor insisted upon by the public. Even when surveillance is instituted, compliance with surveillance regimens is often poor.

Most cost-efficacy models suggest that surveillance is effective and cost effective, although this depends to a great extent on the incidence of HCC.[48], [49], [50], [51], [52]

These results have prompted all professional liver disease societies to recommend surveillance, although the recommended methods vary. In addition, governments in Japan and South Korea have adopted HCC surveillance as public policy.

Surveillance may be by blood tests or by ultrasound or a combination of the two, and some have even proposed MRI as a method of surveillance. The blood tests most often used are alpha-fetoprotein (AFP), des-gammacarboxy prothrombin (DCP) and the L3 fraction of AFP (AFP L3). Combinations of tests have also been used, most commonly AFP and ultrasound. The superiority of this combination over either test alone has not been demonstrated. Unfortunately, the increase in sensitivity that accompanies the use of multiple surveillance tools results in decreased specificity. There are other combinations of biomarkers that have been used, such as the GALAD score,^53^ which uses a combination of AFP, DCP and AFP-L3. However, it is important to note that elevated biomarkers are often associated with more advanced liver disease with a lower likelihood of cure and may therefore be less useful for surveillance. Adding ultrasound may improve performance even further.^54^ Nonetheless, no single method of surveillance has been definitively shown to be superior to any other method in terms of survival. Each suffers from significant false negative and positive rates. In comparative studies some surveillance tests are able to detect more small cancers than the comparator, but whether this translates to improved survival remains unknown.

Professional liver disease societies all recommend surveillance using ultrasound, with or without additional blood tests. However, when ultrasound is not available blood tests alone may be used, but these are likely less effective at detecting curable lesions.

More recently, measurement of methylated cell-free DNA has been proposed as a very sensitive test for early HCC.^55^

There is general agreement about the surveillance interval, which should be 6 months. A 3-month interval is associated with a higher false positive rate^56^ and a 12-month interval is probably associated with lower survival.^57^

There are several important principles that should be observed when instituting HCC surveillance. First, surveillance should be reserved for patients who will benefit from treatment once HCC is diagnosed. Second, the approach should be programmatic rather than relying on individual physicians to decide on management.^58^ This will require participation from health authorities. Third, abnormal surveillance test results that trigger further investigation should be defined. There should be a defined recall procedure that follows a recommended series of investigations in order to minimise the number of tests done, and to decrease the risk of a false positive result and ensure appropriate treatment. For example, the cut-off of AFP or DCP concentration or GALAD score that should trigger ultrasound should be defined for each laboratory. If a mass is seen on ultrasound there should be a defined path of further investigation. The algorithm described in Fig. S1 ^59^ is based on finding a mass on surveillance ultrasound and has been developed to minimise the risk of overdiagnosis and to avoid false negative results.

Thus, early-stage HCC can be identified and cure attempted, but this will be most efficiently and effectively achieved by developing surveillance programmes that are driven by health authorities.

### Diagnosis and staging

The diagnosis of HCC requires either typical radiological features or a biopsy. The typical radiological feature is a lesion that enhances in the arterial phase and is less conspicuous than the surrounding liver in the venous or later phases on multiphase contrast-enhanced CT scan, MRI with contrast, or contrast-enhanced ultrasound. If these features are not present a biopsy is required, usually ultrasound guided. These criteria only apply in individuals with cirrhosis. These characteristics have been incorporated into an algorithm that maps a pathway to HCC diagnosis (Fig. S1).^59^ Intrahepatic cholangiocarcinoma is another cancer that is sometimes seen in cirrhotic livers. However, it does not have the typical radiological features of HCC; thus, biopsy would be indicated if following the aforementioned algorithm. It is important to make the diagnosis correctly, because the management of intrahepatic cholangiocarcinoma is different to that of HCC. It is important that the vascular imaging is done correctly, with an adequate dose of contrast and correct timing of image acquisition after injection of contrast. Failure to adhere to these standards will result in missed diagnoses, particularly in small lesions, which are the most amenable to cure.

Once the diagnosis has been confirmed the next step before offering treatment is to stage the cancer. There are several staging systems that have been proposed, ranging from the standard classification of malignant tumours (TNM) system^60^ to the Barcelona Clinic Liver Cancer (BCLC) system (Fig. S2)^61^ and its derivatives (*e.g.*, the Hong Kong system). While TNM accounts only for tumour characteristics, BCLC takes liver function and performance status into account as well. BCLC has been widely adopted, although there are still adherents to other systems. One advantage of the BCLC staging is that it comes with treatment recommendations appropriate to each stage, something the TNM does not do. The BCLC system was updated recently.^61^

### Providing treatment when cure is possible

Many treatments for HCC are available and their applicability will depend on the characteristics of the tumour, the underlying liver function and health status. If HCC is localised within the liver, locoregional ablative techniques or surgical treatments such as partial hepatectomy or liver transplant are preferred, whereas in the case of extrahepatic spread, a systemic treatment is usually offered. When liver failure exists, locoregional or systemic treatments are contraindicated due to toxicity and the risk of complications, and the only possibility for patients is to consider liver transplantation, but only if the risk of recurrence is low. Choosing the most appropriate treatment for the patient can be complicated and algorithms such as the BCLC algorithm or international guidelines are available to assist clinicians in their choice[61], [62], [63], [64] (Fig. S2).

#### Liver transplantation

Liver transplantation is the treatment of choice as it can cure both HCC and the underlying liver disease. This treatment is recommended as the first-line option for HCC in individuals who have a low risk of HCC recurrence, *i.e*. those within the Milan criteria (a single tumour with a maximum diameter of 5 cm or up to three tumours with a maximum diameter of 3 cm).^65^ Transplantation can also be considered in larger tumours if an attempt to reduce tumour burden to meet Milan criteria (downstaging) is feasible. Vascular invasion by tumour or extrahepatic metastases are absolute contraindications to liver transplant. Currently, individuals with HCC on a background of cirrhosis represent about 30% of the waiting list population in Europe.^62^ Depending on the region involved, living (mostly in Asia) or cadaveric donor liver (mostly in Europe and North America) can be offered. There are major limitations to liver transplantation. The first is the risk of tumour recurrence which, depending on the criteria used for access to transplantation, may range from 6–13%.^66^ Recurrence is associated with a dire prognosis, with greater than 90% mortality. The second is the shortage of organs with a demand for liver grafts greater than the number of available grafts. This imposes constraints in term of indications and results in restrictions to minimise potentially futile transplants The scarcity of donors leads to prolonged waiting times and competition between the different indications for transplantation. The net result is that even in high-resource regions fewer than 5% of individuals with HCC actually receive a liver transplant. This necessitates the use of locoregional treatments to control tumour growth for those on the waiting list, as well as strict rules for graft allocation. For transplantation to be indicated for HCC, the potential recipient must have a greater than 50% probability of survival and less than 15% risk of recurrence at 5 years.^67^^,^^68^ The Milan criteria currently remain the standard for access to transplantation both in Western and Eastern guidelines, but other scores have been proposed to expand the selection of patients for liver transplantation. These include the AFP-French model, University of California San Francisco criteria or the Up-to-7 criteria.[69], [70], [71] In view of the current graft shortage, new approaches have also been proposed, such as the use of marginal cadaveric grafts, split liver transplants or live donor transplants.^68^ This organ shortage should also invite us to develop alternative treatments/strategies, particularly given that liver transplant is only available to a very small portion of the world's population due to the need for trained surgeons, appropriate infrastructure and skill in the use of immunosuppressants. These requirements are not met in all regions.

#### Liver resection

Liver surgery is the first curative option in individuals with early tumours and leads to the best outcomes of any treatment in well selected patients, with 5-year survival rates of 60-80%. Resection has never been compared to no treatment, and since early-stage disease has a good prognosis even without surgery it is not clear whether resection actually decreases mortality. The indication for resection differs depending on local guidelines, but the final decision depends on the volume of the remaining functioning liver after anatomic resection, the surgeon’s experience, the local facilities and also on the underlying liver disease and the stage of fibrosis.[62], [63], [64]^,^^72^ A thorough evaluation of liver function and portal hypertension (a complication of cirrhosis) is crucial before undergoing liver resection. Poor liver function and clinically significant portal hypertension are associated with a higher risk of decompensation and mortality after surgery and depending on severity may be a contraindication. In centres where surgery is frequently performed, research should focus on the development of new indicators to assess liver function and to better identify patients who will benefit from surgery while limiting the risk of complications (accepted goals for post-operative mortality and severe post-surgical morbidity are less than 3% and 30%, respectively). The development of machine learning algorithms combining clinical, imaging, functional and pathological parameters appear particularly interesting in this setting. New surgical techniques have also been developed, mostly in high-resource countries, such as laparoscopy, a robotic approach and “associating liver partition and portal vein ligation of staged hepatectomy” (ALPPS) a staged procedure to improve outcomes in those with marginal liver function.^72^ Moreover, combined preoperative strategies, such as transarterial chemoembolisation (TACE), transarterial radioembolisation (TARE) or portal vein embolisation, are also increasingly used with the aim of allowing resection in a larger number of patients. Adjuvant therapy using immunotherapy is being tested in clinical trials to prevent recurrence in high-risk patients. Even if the outcome of these new strategies is promising, when mentioning liver resection, one must always consider the need to perform these treatments with trained surgeons, in specialised centres, and after appropriate assessment of liver function. Both liver transplantation and liver resection are procedures requiring a high degree of skill, a large team and dedicated nurses, anaesthesiologists, and advanced facilities, which are not widely available in middle- and low-income regions of the world, thus limiting the access to surgery for many patients worldwide.

#### Ablation

When liver resection cannot be performed but the lesion is still not advanced (BCLC stage 0 or A), local ablation is the next option that confers the possibility of cure. Local ablation includes a vast range of techniques and recent advances make it possible to treat tumours of a size that was previously untreatable by local ablation.^73^ Radiofrequency (RFA) and microwave ablation are the standard of care for ablation and have replaced ethanol injection, although ethanol ablation may still have a place in low-resource regions. Classical monopolar RFA is based on generation of an electric current though an electrode inserted into the tumour, and is limited to tumours <3 cm that are not located near a major vessel. RFA works by heating and coagulating the tumour. Large vessels act as a heat sink, reducing the efficacy of the heating. Multibipolar mode can increase the volume of the ablation zone(s), while microwave ablation, which has a higher and faster temperature peak, enables treatment of tumours near large vessels because of a reduction in the heat sink effect. The overall 5-year overall survival rate after RFA varies between 40–70% and the 5-year distant tumour recurrence rate between 58–81% for individuals within the Milan criteria.[74], [75], [76], [77] Laser and cryoablation can also be performed with easy monitoring.^78^ The recent development of irreversible electroporation limits the risk of thermal injury to adjacent structures and eliminates the heat sink effect. However, general anaesthesia using curare and major analgesic drugs are mandatory and can impact patient selection.^79^ All these new approaches may overcome some limitations to local ablation such as location, size and number of tumours. However, poor liver function remains a contraindication to ablation and liver transplantation should be considered for these patients if feasible.^73^ For HCC ≤2 cm, randomised controlled studies comparing liver resection and RFA showed similar overall survival, while RFA was associated with a lower cost.^74^^,^[80], [81], [82], [83], [84], [85] RFA is recommended for first-line treatment in guidelines from Asia but not in guidelines from North America which recommend liver resection for very early HCC. In Europe, guidelines suggest that either RFA or liver resection can be used, depending on the context. For HCC >2 cm, all guidelines recommend resection as a first approach when feasible.[62], [63], [64] However, the choice of treatment should always consider underlying liver disease, expert experience and local facilities. Today, in most major centres, assessment and treatment decisions are made by multidisciplinary teams. Combination with other HCC treatments such as embolisation or local intravascular delivery of drugs may increase efficacy. As commented on for resection, ongoing trials are looking at the use of adjuvant systemic treatment to prevent disease recurrence and improve survival, a major unmet need. Post curative treatment, there is a risk of tumour recurrence due dissemination of tumour clones prior to therapy and/or to the persistent oncogenicity of the underlying abnormal liver. Specific management of the aetiological factors favouring HCC recurrence, such as dietary adjustments for NAFLD-related HCC or complete alcohol abstinence, is required alongside treatment of the HCC itself. Treatment of the underlying viral hepatitis prevents further impairment of liver function, and may even improve it, but the risk of recurrence may not be modified.

#### Transarterial chemoembolisation

TACE is the most commonly used treatment for unresectable, but treatable, HCC. Conventional TACE (cTACE) consists of the infusion of a chemotherapy drug emulsified with lipiodol, an oily radiographic contrast agent, into the artery feeding the HCC, followed by embolisation of the tumour-feeding blood vessels. The most commonly used drugs for cTACE are doxorubicin, epirubicin, cisplatin or miriplatin. Median survival with current criteria should go beyond 30 months and the overall survival at 5 years is around 30%.^86^ TACE with drug-eluting beads (DEB-TACE) can also be used. The embolised microspheres allow for the release of the chemotherapeutic agents in a controlled mode over a 1-week period. Outcome results are similar between cTACE and DEB-TACE. TACE is also used in individuals with early-stage HCC as a bridge to curative treatment, such as liver transplantation or surgery. Ongoing trials are also looking at the impact of adding immunotherapy to TACE or even replacing it. As with liver resection and ablation, TACE can only be performed in individuals with preserved liver function in order to limit the risk of liver failure after the procedure. The risk of hepatic decompensation increases with the number of TACE treatments performed. The use of the ALBI (albumin-bilirubin) score, a measure of liver function, can help in the selection of patients because of its prognostic ability and its potential to identify those patients at higher risk of hepatic decompensation after treatment.^87^ TACE induces tumour necrosis of varying intensity and may be repeated at fixed time points or upon detection of progression. One of the questions that remains is at what point repeating TACE is no longer beneficial or is even detrimental, and if there is a maximum number of TACE procedures that can be performed on the same patient targeted at the same area of the liver.^88^ However, this reflection must consider the chemotherapy agent used and the technique specific to the procedure. Moreover, the recent arrival of immunotherapy will probably lead us to reposition TACE in the therapeutic arsenal. Chemoembolisation is more readily available worldwide, but its application requires the presence of trained practitioners and appropriate facilities.

#### Transarterial radioembolisation

TARE also called radioembolisation is defined as the infusion of radioactive beads into the hepatic artery and delivered to the tumour-bearing area. Although the superiority of TARE *vs.* other forms of therapy has not been demonstrated, it is nonetheless widely used. TARE requires at least two treatment sessions. The first one is a mapping angiogram of the hepatic artery where any arteries passing from the liver circulation to non-target structures can be embolised with coils to prevent radiation damage to these organs. In the same session, 99Tc macroaggregated albumin is injected to calculate the dose of radiation to the tumour and the adjacent liver. This will also show whether there are shunts from the liver to the lung. If these are large the potential for radiation damage to the lung precludes TARE. The second session will consist of the delivery of the radioactive microspheres within the liver tumour (mostly using Yttrium-90). The radiation causes death of the cancer cells over the next 1-3 months. Median survival time after TARE was 17 months for patients at BCLC intermediate stage and 10-12 months for patients at advanced stages with portal vein invasion. Several studies have shown no survival benefit of TARE over systemic therapies in individuals with advanced HCC.^89^^,^^90^ However, TARE may be more valuable in individuals with borderline resectable HCC as it controls the tumour but also induces substantial hypertrophy in the liver lobe contralateral to the target, or as a bridge to liver transplant.^91^^,^^92^ Moreover, a recent trial demonstrated the importance of personal dosimetry in increasing the response to treatment.^93^ Accessibility to TARE requires close collaboration between the interventional radiologist, nuclear medicine specialists, radiopharmacists, and physicists. These specialists are not present in all hospitals.

#### External beam radiation therapy

Historically, liver external beam radiation therapy (EBRT) was not used because of the risk of radiation-induced liver disease.^94^ However, improvements in the understanding of the liver parenchyma’s radiation dose tolerances have led to reconsideration of EBRT in the context of other local treatment options in individuals with compensated cirrhosis. The recent use of 3D-conformal radiotherapy, intensity modulated radiotherapy, stereotactic-body radiotherapy and proton beam radiotherapy have improved the efficacy of EBRT by increasing the radiotherapy dose to the tumour while simultaneously reducing the dose to the surrounding normal liver parenchyma.^95^ Robust trials of radiotherapy are still pending, but preliminary data are promising across BCLC stages.^96^ Comparative trials are currently ongoing to define optimal sequencing of stereotactic-body radiotherapy alone or in combination with other treatments modalities, such as TACE or systemic therapy, considering patient-reported outcomes, costs and efficacy outside of expert centres.

#### Systemic therapies

Systemic therapies are indicated for patients presenting with advanced disease, or for those presenting with intermediate stage disease that has progressed on locoregional therapy. After nearly a decade of negative phase III trials, there have been major advances since 2016, first with tyrosine kinase inhibitors (TKIs) and more recently with immunotherapy (Table 2).[97], [98], [99], [100], [101], [102], [103], [104], [105] Indeed, atezolizumab-bevacizumab, a combination of an immunotherapy agent and an angiogenesis inhibitor, is the new standard of care for advanced HCC as it was associated with a significant improvement in survival (67% at 12 months) compared to sorafenib (55% at 12 months), the first approved TKI in 2007.^106^^,^^107^ A recent study also showed an improvement in survival with the combination of durvalumab-tremelimumab (16.5 months) compared to sorafenib (13.8 months). However, these combinations of treatments are not yet available everywhere and TKIs remain the standard of care for some countries. All forms of therapy for HCC are expensive, but systemic therapy requires ongoing expense, possibly over many months, compared to the one-time expense implied in other treatments. Therefore, careful consideration of the cost-efficacy equation is warranted for systemic therapy, particularly in low-income regions.

**Table 2:** **Phase III trials of systemic therapies**.

Study	Design of the study	Median overall survival
**First-line treatments**		
Llovet *et al.* NEJM 2008^99^	SHARP study Phase III RCT Sorafenib (n = 299) *vs.* placebo (n = 303)	Sorafenib 10.7 monthsPlacebo 7.9 months
Kudo *et al.* Lancet 2018(100)	REFLECT study Phase III RCT Sorafenib (n = 476) *vs.* lenvatinib (n = 478)	Sorafenib 12.3 months Lenvatinib 13.6 months
Finn *et al.* NEJM 2020(106)	IMBRAVE 150 study Phase III RCT Atezolizumab/bevacizumab (n = 336) *vs.* sorafenib (n = 165)	Sorafenib 13.4 months Atezolizumab-bevacizumab 19.8 months
Yau *et al.* Ann of Oncol 2020(103)	CHECKMATE 459 Phase III RCT Nivolumab (n = 371) *vs.* sorafenib (n = 372)	Sorafenib 14.8 months Nivolumab 16.4 months
Abou-Alfa *et al.* NEJM 2022(105)	HIMALAYA study Phase III RCT Tremelimumab/durvalumab (n = 393) *vs.* durvalumab (n = 389) *vs.* sorafenib (n = 389)	Tremelimumab/durvalumab 16.4 months Durvalumab 16.6 months Sorafenib 13.8 months
**Second-line treatments**		
Bruix *et al.* Lancet 2017(98)	RESORCE study Phase III RCT Regorafenib (n = 379) *vs.* placebo (n = 194)	Regorafenib 10.8 months Placebo 7.8 months
Abou-alfa *et al.* NEJM 2018(101)	CELESTIAL study Phase III RCT Cabozantinib (n = 470) *vs.* placebo (n = 237)	Cabozantinib 10.2 months Placebo 8.0 months
Zhu *et al.* Lancet 2019(102)	REACH-2 study Phase III RCT Ramucirumab (n = 470) *vs.* placebo (n = 237)	Ramucirumab 7.6 months Placebo 7.3 months
Finn *et al.* J Clin Oncol 2020(104)	KEYNOTE-240 study Phase III RCT Pembrolizumab (n = 279) *vs.* placebo (n = 134)	Pembrolizumab 13.8 months Placebo 10.6 months

With the increasing clinical impact of systemic therapy, the most appropriate treatment sequence as well as the appropriate timing to shift from locoregional to systemic or combination therapy remains unclear.^97^^,^^108^^,^^109^ Ongoing trials looking at the benefits of combining locoregional with systemic therapy in selected patients will probably expand the future indications for systemic therapy. However, real life data are needed as patients included in the studies were carefully selected and are probably not representative of the larger population.

Thus, treatment is available for all but the most terminal cases of HCC. Treatment is expensive and requires a large team with different expertise and substantial institutional resources.

#### Future of HCC treatment

HCCs are characterised by considerable phenotypic and molecular heterogeneity. Treating HCC is particularly challenging because of the underlying liver disease and the specific biological mechanisms that lead to cancer development and progression, which may further impact the response to treatment. Next-generation sequencing has facilitated the discovery of the main signalling pathways that are altered in HCC. This has allowed for the classification of liver cancers according to their genotypes and the underlying causes of liver disease – it is possible that treatment choice might be dictated by these classifications in the future. The combination of tumour and non-tumour prognostic genetic signatures, as well as histological and clinical features may allow for accurate prediction of prognosis in individual patients, as well as enabling so-called “precision medicine”. However, further translational studies on clinical samples will be required before such personalised approaches become a clinical reality.^110^

## Recommendations

The recommendations that follow are stratified by the availability of resources in any particular region. The regions are defined as follows:

• *Low-resource regions*: These are defined as regions where surgical treatment of HCC is only occasionally available, TACE is not available, nor is liver transplantation and systemic therapy is unaffordable. Ultrasound is available in urban centres only. Laboratories are capable of measuring AFP.
• *Medium resource regions*: These are defined as regions where most HCC therapies are available except for liver transplantation. Ultrasound is widely available. All routine laboratory tests are available including AFP, and the tests used in at least one of the fibrosis panels that require more than the APRI or FIB-4 blood tests (*e.g.*, alpha-2 macroglobulin, haptoglobin, apolipoprotein A1, GGT for the Fibrotest or procollagen III amino-terminal peptide, hyaluronic acid or tissue inhibitor of matrix metalloproteinase 1 for the ELF score).
• *High-resource regions:* These are defined as regions where, in addition to the conditions listed for medium resource countries, transplantation and all licenced systemic therapies are available.

The initial set of recommendations represents the ideal, likely available only in high-resource regions. This is followed by modifications to the recommendations where necessary for medium- and low-resource regions.

### Primary prevention of HCC

#### Management of hepatitis B

• **Neonatal hepatitis B vaccination consisting of three doses of vaccine, best provided as a multivalent vaccine with other childhood vaccines, plus the addition of hepatitis B immune globulin for the babies of infected mothers. Pregnant hepatitis B-infected mothers should be treated with tenofovir to decrease viral load and reduce the risk of transmission to the baby.**

This should be publicly funded. Adolescent vaccination, if used, should not replace universal neonatal vaccination. Guidelines for vaccination of older age groups and for post-exposure prophylaxis promulgated by World Health Organization should also be adhered to.

• **In regions where universal neonatal vaccination has not been or has only recently been instituted and which are considered as high prevalence regions, there should be one-time screening of the general population for HBsAg, with treatment of those who are positive and who have active disease. The family members of positive individuals should also be screened as well as people who would need protection through vaccination.**
• **Treatment of HBsAg-positive individuals with active disease should be according to North American, European or Asian guidelines as appropriate.**

#### Management of hepatitis C

• **In Western countries there should be government backed one-time screening for hepatitis C (anti-HCV assay) in the population born between 1945 and 1975 or 1985, depending on the population. Positive individuals should be tested for active disease with HCV RNA and if positive should be referred for treatment.**

Positive individuals should be assessed for the presence of liver disease and cirrhosis. In regions with high HCV prevalence, screening strategies may vary. In some it might be worth screening the entire population who were born before the introduction of disposable needles and syringes. All patients who were transfused with blood or blood products prior to 1990 should also be screened. Studies to determine whether this would be cost effective in the local region would help with decision-making.

• **Test and treat programmes should be instituted for difficult to reach populations.**

Such programmes can utilise rapid diagnostic tests. Patients who are HCV RNA-positive are entered into treatment programmes immediately.

• **Those who are infected with hepatitis C should be treated.**

Current treatment can be used for all genotypes and severities of liver disease. The newest generation of direct-acting antiviral agents, which cure hepatitis C in 95% of cases, are available at a cost of $60 or less for a course of treatment.^111^

#### Management of NAFLD

The underlying causes of NAFLD do not lend themselves to easy solutions. A detailed discussion of ways to address this are beyond the scope of this document. However, potential strategies proposed by the World Bank are listed in Table 3.^11^ The strategies involved require government intervention. Any approach to managing NAFLD as a means to reduce HCC incidence will involve a whole-of-society approach to better nutrition and greater physical activity. The World Bank has produced a document that goes into the problem of obesity and its solutions in great detail.^11^ The reader is referred to this document.

**Table 3:** **Strategies to reduce obesity**.

Intervention	Strategy	Outcome
Fiscal policy	Tax on sugared beverages	Effective (*e.g.* Mexico)
	Tax on fat in unhealthy foods	Effective (*e.g.* Kerala, India)
	Tax on processed foods	Uncertain effect (*e.g.* Mexico)
Regulatory policy	Front of package product labelling (*e.g*. to identify ultra-processed food and drink, to identify healthy food and drink, to provide a grading system for labelling)	Potential impact
	Regulation of marketing and sales of unhealthy foods (*e.g*. to children)	Some impact
	Eliminate subsidies on unhealthy foods (*e.g.* sugar, palm oil)	Uncertain effect
Reduce sedentary activity	Encourage walking by urban design	Theoretical
Agricultural/food systems		Little known

#### Management of alcohol-related liver disease

The global increase in alcohol consumption is also difficult to address. A detailed discussion of possible strategies is beyond the scope of this document. For a more detailed discussion the reader is referred to the World Health document, Global Status Report on Alcohol and Health from 2018.^12^ Some strategies that have been tried are listed in Table 4. In addition, medical practitioners should systematically inquire about alcohol use and provide advice to those who either consume excess alcohol, or who do not consume in excess, but have other risk factors for HCC. However, primary prevention, the diagnosis of liver disease and assessment of its severity as well as surveillance has to be conducted by primary healthcare workers, family practitioners, nurse practitioners and the like. The approach to hepatitis C in Egypt, and Japan, and hepatitis B in Korea and Taiwan are examples.

**Table 4:** **Alcohol control strategies**.^12^

Intervention	Examples
National leadership	Written alcohol control policies
	Awareness-raising activities
Treatment coverage for alcohol use disorders	Proportion of patients in care for excess alcohol largely unknown
Government support for community action	Provision of educational information
	Training programs
	Dissemination of data
	Research studies
Drinking and driving	Sobriety checkpoints
	Drunk driving policies
Regulating alcohol availability	Raising legal age for purchase
	National control of production and sale
	Restrictions on outlet density and site (*e.g.*, near schools)
	Restrictions on drinking in public
Restrictions on alcohol advertising	
Pricing	Alcohol taxes and/or control of pricing
Warning messages	Pregnancy, other health warnings
Addressing informal and illicit alcohol production and consumption	

### Secondary prevention

#### Diagnosis of liver disease

• **ALT and GGT tests should be part of the annual medical check-up by family physicians.**

For those who do not attend regular medical appointments, or who do not have access to routine medical check-ups, ALT should be a part of the work-up at initial presentation. Routine testing for GGT is recommended by the Lancet-EASL commission^112^ and other commissions and is an important strategy to identify NAFLD.

• **Any abnormal ALT or GGT requires investigation to determine the cause.**
• **If liver disease is diagnosed the degree of liver function and fibrosis should be assessed using the methods described earlier.**

#### Surveillance

• **Surveillance programmes for HCC should be developed by local health authorities.**

These should include consideration of target populations, surveillance methods and recall procedures. Surveillance for HCC is not appropriate if curative therapy is not possible because of advanced liver disease or other reasons, or if not available in the local healthcare system.

• **Individuals with cirrhosis or stage 3 fibrosis or long-standing chronic hepatitis B should be assessed for HCC risk using one of the methods described earlier.**

Each risk score has a cut-off, below which surveillance is not required. All others should undergo 6-monthly surveillance. The methods used for surveillance depend on local resources. If ultrasound is used this should be high-quality ultrasound. Obtaining a high-quality ultrasound picture in individuals with cirrhosis or NAFLD is difficult. It is therefore recommended that ultrasound surveillance be performed by specially trained technicians (similar to the requirement in the USA for specific training to perform ultrasound in pregnancy). Ideally, surveillance ultrasound should be performed in expert centres, but where liver disease is common this is probably not practical.

• **If using blood tests, the measurement of AFP is a minimum but, if available, the GALAD score blood tests should be used.**

If blood tests are used, depending on the local laboratory, cut-off levels should be set that would trigger further investigation. Depending on resources and on available skill this can be by ultrasound or by CT scan or MRI. Six-monthly intervals are recommended. If surveillance relies on blood tests alone it is important to recognise that a variable proportion of individuals with HCC will be negative for one or more of all the usual markers. Furthermore, outcomes are likely to be less good because biomarkers tend to only become elevated with later stage disease.

#### Diagnosis

• **The diagnostic algorithm described earlier should be followed.**

Assuming that a small lesion has been seen on ultrasound, either in association with an elevated AFP or not, if the liver is cirrhotic this is highly likely to be HCC or a related cancer such as cholangiocarcinoma, or one of the mixed hepatocellular/cholangiocellular carcinomas. The only other common lesion might be a haemangioma which should be easily diagnosed on ultrasound.

• **The diagnosis should be confirmed either by radiology according to the algorithm described in**Fig. S1**, or by biopsy if following the algorithm does not result in a clear diagnosis.**

A fine needle core biopsy carries least risk. As a note of caution, sometimes histological interpretation of very small HCCs can be misleading and can be described as normal or dysplastic. This is because very early HCCs can resemble normal liver tissue except for subtle changes such as an increase in the cellularity of the liver cords. If expertise in liver pathology is not available, cases should be referred for expert interpretation. For lesions >3 cm in diameter there is seldom much diagnostic uncertainty.

• **If the diagnosis is confirmed the lesion should be staged according to the BCLC system.**

Although other systems can be used the BCLC is preferred in order to ensure uniformity across different jurisdictions.

#### Treatment

• **Ideally, treatment should be made available in expert centres or networks where all the necessary medical specialties are represented.**

This includes hepatology, hepatobiliary surgery, oncology, radiology, interventional radiology, pathology, radiation oncology, and other specialities and support staff and facilities necessary to provide all the recognised HCC treatments, including liver transplant, hepatic resection, systemic therapy, local ablation and chemoembolisation. In some instances, after a tumour board has made a recommendation, treatment can be administered locally (this is particularly applicable to systemic treatment). Centres that only see a few cases/year are unlikely to have the necessary expertise and facilities. Furthermore, centres of excellence are more likely to have skilled support personnel, such as specialised nurses and social workers.

• **The treatments and indications recommended by scientific guidelines should be followed.**

The treatments suggested in the scientific guidelines are supported by extensive research that confirms their efficacy. Some treatments such as EBRT or combination treatment still require validation. Research in radiotherapy is ongoing and new results are expected in the near future.

## Middle resource regions

### Primary prevention of HCC

#### Management of underlying causes

• **The recommendations regarding hepatitis B and C described above should be followed.**

These regions should institute universal hepatitis B vaccination. In high prevalence areas the unvaccinated population should be screened for hepatitis B, and all carriers or those with active disease should be vaccinated or treated. Hepatitis B DNA testing should be available to identify those with active viral replication. Hepatitis C screening programmes should be instituted. The target population will vary according to local demographics and local prevalence. All who are positive should be treated with modern direct-acting antiviral agents.

• **Governments should develop programmes to combat obesity and alcoholism as described elsewhere.**

Since the burden of obesity is greatest in middle-income countries and is passing more and more to the poor, such programmes are vitally important to prevent multiple health problems in the future. Such programmes have to be government backed to enable the broadest possible coverage.

### Secondary prevention

#### Diagnosis of liver disease

• **The recommendations described above should be followed.**

ALT and GGT should be routinely tested for as described previously. All abnormal results require further investigation. Fibrosis should be assessed in everyone with liver disease as described above. All these recommendations are well within the reach of middle resource regions.

#### Surveillance

• **The recommendations described earlier should be followed.**

Surveillance with ultrasound and/or AFP or the GALAD combination of tests should be offered to all persons with cirrhosis or a risk score high enough to warrant surveillance. Training programmes for ultrasonographic surveillance should be developed to ensure best results.

#### Diagnosis and staging

• **The recommendations described earlier should be followed.**

The American Association for the Study of Liver Disease (AASLD) algorithm for investigation should be followed. The BCLC algorithm should be followed to stage the cancer.

#### Treatment

• **Ideally, treatment should be made available in expert centres or networks.**

Even if liver transplantation is not available treatment should be offered only in expert centres and not in centres that deal with only a few cases/year.

• **The treatments and indications recommended by scientific guidelines should be followed.**

## Low-resource regions

### Primary prevention of HCC

#### Management of underlying causes

• **The recommendations regarding hepatitis B, D and C described above should be followed.**

These regions should institute universal hepatitis B vaccination. This measure is likely to have the single largest impact on the incidence of HCC. In high prevalence areas the unvaccinated population should be screened for hepatitis B, and all those with active disease should be treated. If DNA testing is not available, ALT can be an imperfect but useful surrogate for hepatitis B DNA in HBsAg-positive individuals. An elevated ALT in someone who is HBsAg-positive should be treated regardless of HBeAg status. Tenofovir disoproxil fumarate and entecavir are no longer patent protected, and are therefore inexpensive, although tenofovir alafenamide remains on patent. A year’s course of generic tenofovir can be obtained for $48 or less.^9^

Hepatitis C screening programmes should be instituted. The target population will vary according to local demographics and local prevalence. All who are positive should be treated with modern direct-acting antiviral agents. These are also now available at low cost.

• **Governments should develop programmes to combat obesity and alcoholism as described elsewhere.**

The burden of obesity is falling more and more to low-income regions. Therefore, programmes to minimise the risks for obesity are necessary to prevent multiple health problems in the future. Such programmes have to be government backed to enable the broadest possible coverage.

### Secondary prevention

#### Diagnosis of liver disease

• **The recommendations described above should be followed.**

ALT and GGT should be routinely tested for as described previously. All abnormal results require further investigation. Fibrosis should be assessed in everyone with liver disease as described above. If fibrosis panel testing and measurement of liver stiffness by transient or shear-wave elastography are not available, the best available measures are ultrasound (showing an irregular outline to the liver and increased echogenicity), or a platelet count below normal in the absence of haematological disease. Non-invasive serologic tests such as those listed in Table S4 can be used.

#### Surveillance

• **In urban areas surveillance with ultrasound and/or AFP should be instituted for those who have been diagnosed with cirrhosis.**

Risk scores are of little use in those with cirrhosis, who should all undergo surveillance. However, surveillance should only be undertaken if facilities exist to treat small HCCs with local ablation. In most low-resource regions this will mean alcohol injection. In rural areas it is probably impractical to have a traveling ultrasound machine and technician. If so, surveillance could be with AFP alone, but successful early detection and long-term disease-free survival will be suboptimal.

#### Diagnosis of HCC and staging

If CT scanning or MRI is available these should be used for diagnosis. These techniques are adequate for diagnosis if the lesion is 1 cm or greater. Diagnostic work-up is required if a nodule is >10 mm. A specific pattern of contrast enhancement (contrast uptake in the arterial phase followed by washout) confirms the diagnosis. If the typical pattern is not seen a biopsy is required for unequivocal diagnosis.

#### Treatment

The only effective treatment that might be available in low-resource regions is local ablation, or occasionally hepatic resection in individuals with excellent liver function. However, the decision to perform a resection has to consider the possibility of recurrence, and only those with a low risk of recurrence and with expected outcomes that would be superior to other alternative approaches should be offered resection. If recurrence were to occur it is unlikely that any further treatment would be available.

## Expert and network centres

Many aspects of HCC management require high levels of expertise. Ultrasound surveillance requires a high degree of technical skill, both in relation to its performance and interpretation. CT scan or MRI need to be performed with the appropriate doses of contrast medium and appropriate timing of acquisition sequences, as well as expert interpretation of the images. Liver biopsy of early lesions also requires expertise and experience. Of course, liver transplantation is technically and clinically demanding, but expertise is also required to properly deliver other forms of treatment as well, such a TACE, or local ablation with alcohol, RFA or microwave techniques. The advanced level of skill required for these different approaches suggest that the best outcomes are likely to be achieved in expert centres. These should be established wherever the demand is sufficiently high and health authorities should suggest referral to these centres for individuals diagnosed with HCC.

## Research

The literature on HCC, unfortunately, is replete with retrospective and uncontrolled studies that have not led, with a few exceptions, to health authorities setting up programmes or issuing regulations or even guidance. Until the pharmaceutical industry started testing therapeutics for HCC, randomised controlled trials were few and far between. Thus, there is a lot of incomplete work and much opportunity to establish ground-breaking studies.

### Epidemiology

Although there are estimates in very many countries of the incidence and prevalence of viral hepatitis, the quality of these estimates varies widely. Countries should undertake routine surveillance at intervals to determine whether the underlying causes, namely hepatitis B or C are increasing or decreasing in their region and to determine the demographics of the infected populations.

### Surveillance

A randomised controlled trial of surveillance *vs.* no surveillance is no longer possible. However, it is possible to perform a randomised controlled trial comparing different methods of surveillance. As newer surveillance tools are developed they should be tested against older tools. Ideally, the endpoint of such studies should be survival, but this could lead to impractically long studies given that surveillance will find early treatable lesions, so surrogate markers, such as time to first recurrence in the treated area or transition to a more advanced disease stage, will have to be used.

There have been many cost-efficacy analyses of surveillance, but none have modelled the modern approach to HCC management. Thus, new modelling studies are needed.

### Diagnosis

The AASLD-defined diagnostic radiological criteria have not been compared prospectively to alternative pathways such as the liver imaging reporting and data system (Li-RADS) diagnostic algorithm in terms of performance characteristics, such as sensitivity, specificity, negative and positive predictive values, and false positive and negative rates. Both are accepted strategies, so a direct comparison would meet the criteria of ethical balance and clinical equipoise. While the diagnostic requirements outlined by AASLD and EASL divide nodules characterised by CT or MR as definitively benign, definitively HCC or non-diagnostic thus requiring a biopsy, Li-RADS stratifies the imaging observations according to the probability of a nodule being an HCC. If the nodule does not meet the criteria for definitive HCC, Li-RADS recommends follow-up because there is a lower probability of HCC. However, HCC may be present in 20–40% of such non-diagnostic nodules. Therefore, this strategy results in a delay in diagnosis before radiological changes allow for a definitive diagnosis of HCC and may result in a poor long-term outcome. Thus, research is needed to define the proper approach and validity of the Li-RADS recommendation.

With regard to biopsy, the availability of tissue has the potential to allow for research into pathological mechanisms or new biomarkers for diagnosis or prognosis or response to treatment. However, biopsy does come with risks of needle track seeding^113^ or bleeding. Thus, if diagnosis has been confirmed by imaging, biopsy sampling should be considered as research and should be performed with consent under the usual research ethics conditions.

### Treatment

There are numerous studies that could be undertaken. However, recruitment to studies is sometimes difficult. To date individual pharmaceutical companies have conducted studies without reference to what other companies might be doing. However, volunteers for research studies are a precious resource and new study designs such as adaptive trials, umbrella designs or platform designs would speed up investigations of newer therapies. It is also to the pharmaceutical companies benefit to participate in adaptive trials because ineffective drugs might be identified without the need for a large randomised controlled trial and the accompanying expense. Therefore, it is recommended that networks of investigators be developed and that these networks propose adaptive studies whereby research participants’ results can be used for multiple studies. This requires uniformity of inclusion and exclusion criteria in order to standardise the study population.

Studies in early HCC may not be able to have survival as an endpoint because of the length of follow-up required until death from HCC, or because, if the treatments are successful, death from HCC does not occur. These studies may need to use surrogate markers of treatment efficacy such as time to recurrence or time to progression or time to deterioration of liver function and/or performance status. Such studies might be confounded by the development of second primaries remote from the location of the first that was treated in the study.

For studies in later HCC (*e.g.*, BCLC B or C), survival should be the endpoint for all treatment studies, since surrogate markers of efficacy, particularly those that rely only on anatomical changes (recurrence or progression) do not take liver function into account. All treatment studies using new drugs should, in addition to having survival as an endpoint, explore the criteria that define whether an individual is a responder or non-responder.

Finally, the current system of reporting of adverse events is not appropriate for studies in liver cancer. Since hepatotoxicity is one of the most common reasons for a drug not making it to market, and given that liver test abnormalities are frequent in HCC, the widely used classification system CTCAE (Common Terminology for Adverse Events) needs to be redesigned specifically for HCC to better represent the adverse events that can occur in liver disease in general and liver cancer in particular.^114^ The classification in use at present does not represent the current understanding of liver disease and its complications.

An important part of the research into the benefit of treatment of HCC, particularly in patients in whom cure is not likely, is the assessment of quality of life and this should now be included in all trials, particularly those of systemic treatment in more advanced HCC. Research should also explore the balance between the possibility of survival benefit and potential deterioration in quality of life.^115^ Quality of life scales and patient-reported outcomes already exist but these should be refined to ensure that they are applicable across societies with heterogeneous cultures and social values.

## Conclusion

HCC is a rampant cancer with generally poor outcomes, but the data and strategies presented in this document, if implemented, will reduce the incidence of HCC and will likely also reduce the death rate from this disease. Pharmaceutical companies could help by reducing the costs of drugs used to treat HCC in low-income regions.

Additional information pertaining particularly to Europe can be found in these documents:

https://digestivecancers.eu/publication/white-paper-liver-cancer-no-patient-left-behind/

https://digestivecancers.eu/publication/the-cost-of-cancers-of-the-digestive-system-in-europe/

## Financial support

The authors received no financial support to produce this manuscript.

## Authors’ contributions

Manon Allaire – Concept, writing and editing. Jordi Bruix – concept, reviewing document. Marko Korenjak – manuscript review. Sarah Manes - manuscript review. Zorana Maravic – manuscript review. Helen Reeves - manuscript review. Riad Salem - manuscript review. Bruno Sangro – concept and manuscript review. Morris Sherman – concept, writing and editing.

## Conflict of interest

Please refer to the accompanying ICMJE disclosure forms for further details.
