# Supplementary material for: What to do about hepatocellular carcinoma: Recommendations for health authorities from the International Liver Cancer Association
Source: JHEP Rep. 2022 Sep 8;4(12):100578. doi: 10.1016/j.jhepr.2022.100578 (PMC9638834; doi:10.1016/j.jhepr.2022.100578)
Supplement: Multimedia component 1 [file mmc1.pdf]

What to do about hepatocellular carcinoma: Recommendations for health authorities from the International Liver Cancer Association

Manon Allaire, Jordi Bruix, Marko Korenjak, Sarah Manes, Zorana Maravic, Helen Reeves, Riad Salem, Bruno Sangro, Morris Sherman

Table of contents

Fig. S1..2

Fig. S2..3

Table S1..4

Table S2..5

Table S3..6

Table S4..7

Table S5..8

Fig. S1. Algorithm for the investigation of a liver mass found on surveillance. This algorithm is probably only feasible in high and middles resource regions (adapted from (59))

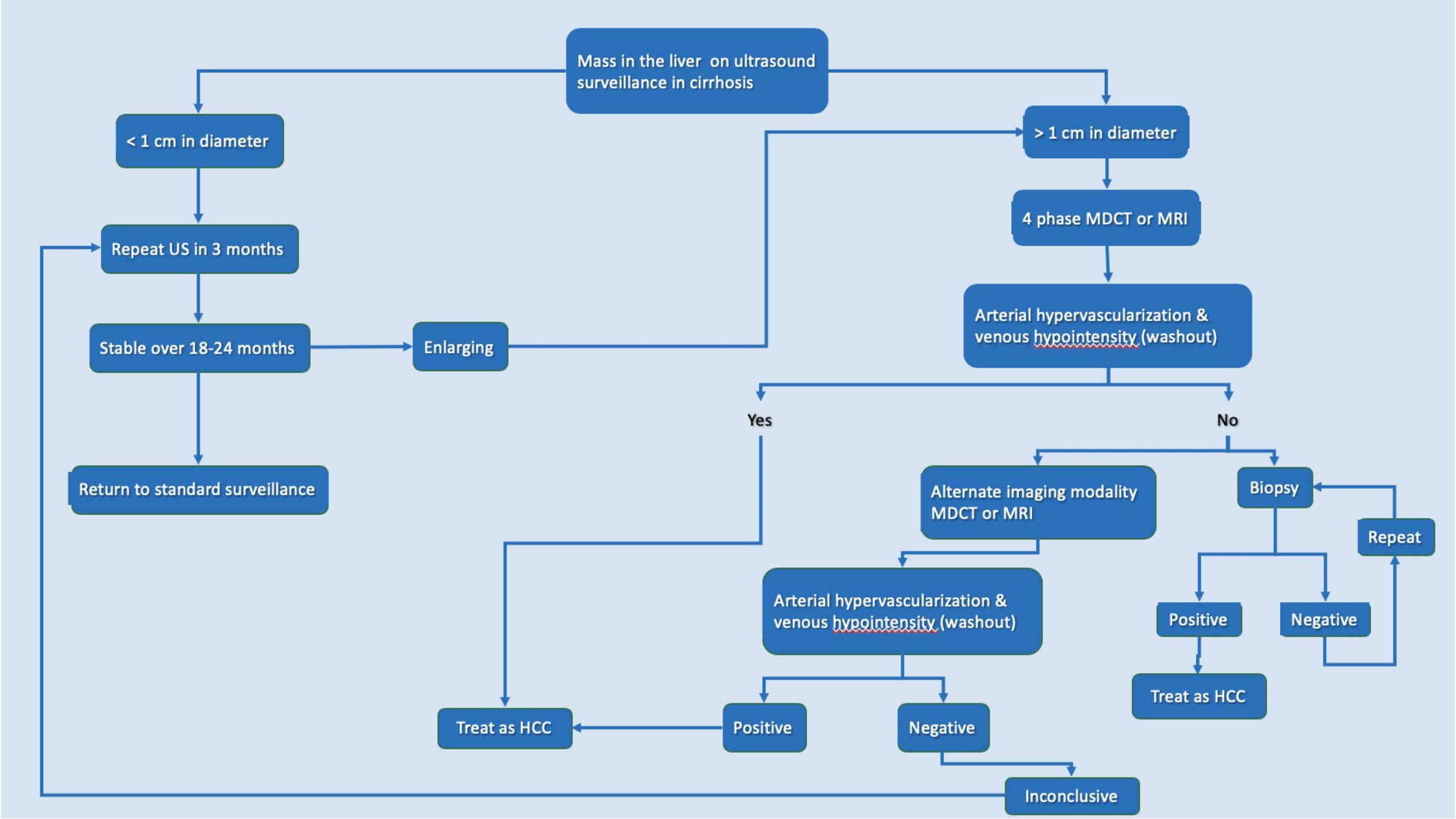

HCC – hepatocellular carcinoma
US – ultrasound
MDCT – multidetector computed tomography
MRI – magnetic resonance imaging

Fig. S2. The BCLC Staging system and suggested treatment options (Adapted from (61))

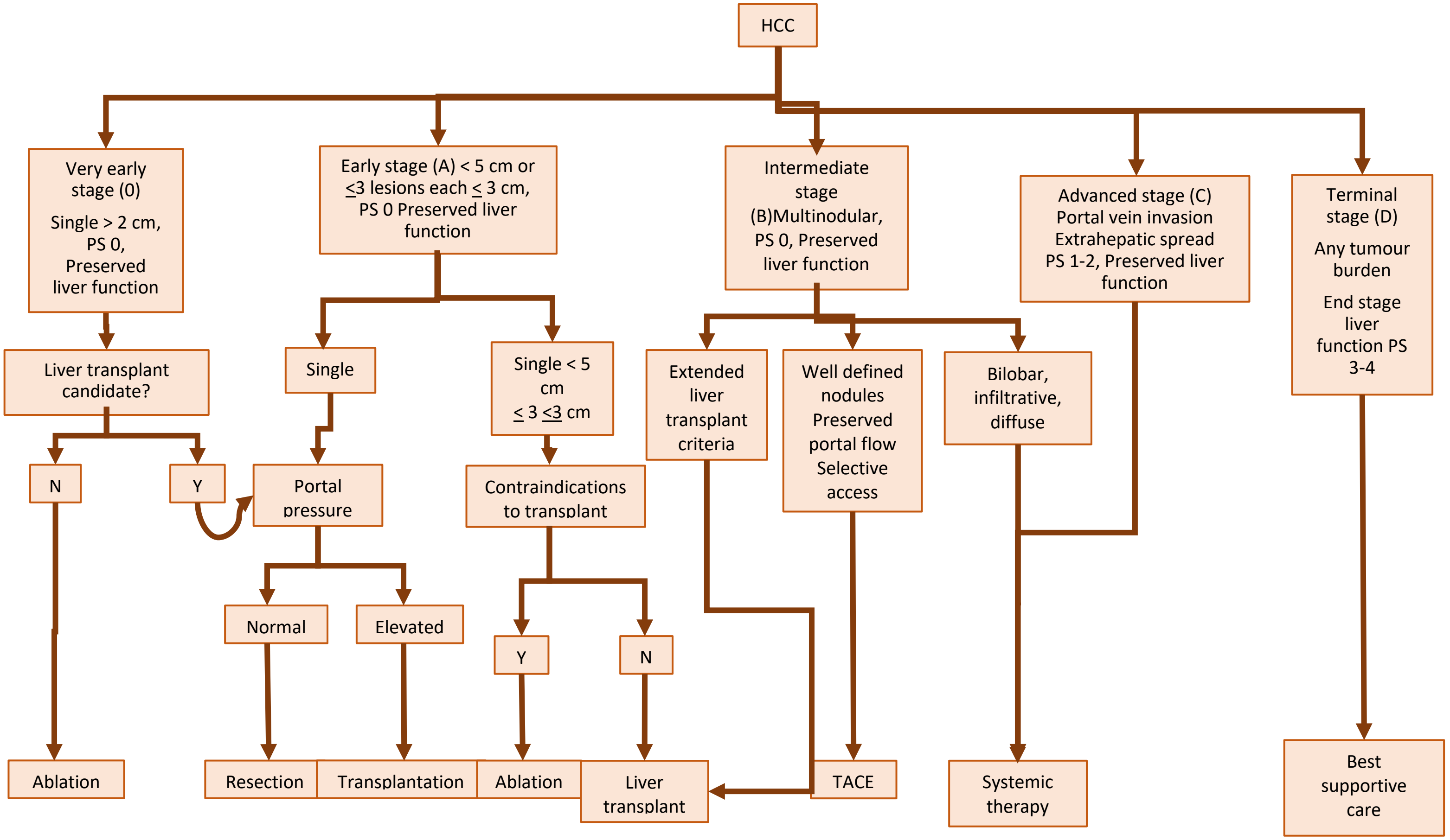

HCC – hepatocellular carcinoma
PS – performance score

Table S1. Incidence of hepatocellular carcinoma (6)

Region	Incidence/100,000
Global	12.8
Asia Pacific high income	26.4
East Asia	24.3
Western sub-Saharan Africa	16.9
South East Asia	13.9
Central sub-Saharan Africa	16.9
Oceana	10.8
East sub-Saharan Africa	10.3
Southern sub-Saharan Africa	8.6
Western Europe	9.0

Table S2. Epidemiology of chronic hepatitis B (9)

WHO* region	Best estimate of prevalence (% infected)	Best estimate of number of infected (millions)
African region	6.1	60
Region of the Americas	0.7	7
Eastern Mediterranean region	3.3	21
European region	1.6	15
South East Asia region	2.0	39
Western Pacific Region	6.2	115

*World Health Organization

Table S3. Epidemiology of chronic hepatitis C (9)

WHO* region	Best estimate of Incidence /100,000	Best estimate of number of infected (thousand)
African region	31	309
Region of the Americas	6.4	63
Eastern Mediterranean region	62.5	409
European region	61.8	565
South East Asia region	14.8	287
Western pacific region	2.0	111
Total	23.7	1751

*World Health Organization

Table S4. Non-invasive blood test panels for fibrosis (25–31)

Test panel	Component blood tests
APRI (25)	AST, platelets
Fib-4 (25)	Age AST, ALT, platelets
Fibrotest (26)	Age, gender, bilirubin, GGT, ∝2M, Apo-A1
Hepascore (27)	Age, gender, bilirubin, GGT, ∝2M
Fibrometer (28)	Age, AST, platelets, prothrombin index, urea
Forns index (29)	Age GGT, platelets, prothrombin, cholesterol
ELF panel(30)	Age Hyaluronic acid, TIMP-1, PNPIII
NAFLD fibrosis score (31)	Age BMI, diabetes, ASL/ALT, platelet count, albumin

APRI - AST to platelet ratio index; NAFLD – nonalcoholic fatty liver disease; ELF – Enhanced Liver Fibrosis
AST – aspartate aminotransferase. ALT alanine aminotransferase, GGT- gamma glutamyl transferase,
∝2M – alpha 2 macroglobulin, TIMP-1 – tissue inhibitor of metaloproteinase-1,
PNPII – N-terminal procollagen peptide 3, BMI- basal metabolic index

Table S5. Scores to assess HCC risk

Risk Score	Population	Components
Reach B (34)	Asian hepatitis B carriers	Age, ALT, HBeAg+/-, gender, HBV DNA
Reach BII (42)	Asian hepatitis B carriers	Age, ALT, HBeAg+/-, gender, HBsAg concentration
CU-HCC (43)	Asian hepatitis B carriers	Age, albumin, HBV DNA, cirrhosis+/-
GAG-HCC (35)	Asian hepatitis B carriers	Age, gender, HBV DNA, cirrhosis+/-, core promoter mutation
PAGE B (36)	Caucasian hepatitis B carriers	Age, gender, platelets
CAGE B (44)<br>SAGE B	Caucasian hepatitis B carriers after 5 years of follow-up	Age, baseline cirrhosis+/-,LSM[1]<br>Age, LSM (baseline cirrhosis status unknown)
Toronto (37)	Cirrhosis of various etiologies	Age, etiology, gender, APRI score
Adress (39)	Hepatitis C cirrhosis	Age, diabetes+/-, gender, Child-Pugh score[2], Race
HALT C Trial Group (40)	Hepatitis C cirrhosis	Age, Race, ALP, esophageal varices+/-, ever smoked+/-, platelets
ANRS CO12 CirVir Study (41)	Hepatitis C	Age, alcohol excess+/-, platelets, GGT, lack of SVR[3]
aMAP (38)	All liver disease	Age gender, ALBI score[4], platelets
PLAN B (45)	Chronic hepatitis B	Cirrhosis, age, platelets, antivirals, sex, ALT, HBV DBA, albumin, bilirubin, HBeAg

[1]LSM-liver stiffness measurement.

[2]Child-Pugh score – used to assess liver function.

[3]SVR – sustained virological response (cured hepatitis C). [4]ALBI score – used to assess liver function.
